# Exploring the Influence of Dark Triad and Light Triad Traits on Sport Sciences Students

**DOI:** 10.3390/medicina60081269

**Published:** 2024-08-06

**Authors:** Sermin Agrali Ermis, Ayse Hazal Boyanmis, İnci Kesilmiş, Turhan Toros, Emre Bulent Ogras, Manolya Akın, Cenk Temel, Alper Cenk Gurkan, Yesim Karac Ocal

**Affiliations:** 1 Faculty of Sport Sciences, Aydin Adnan Menderes University, Aydin 09010, Türkiye; 2 Faculty of Sport Sciences, Mersin University, Mersin 33110, Türkiyeemrebulentogras@gmail.com (E.B.O.); 3 Department of Coaching Education, Faculty of Sport Sciences, Mersin University, Mersin 33110, Türkiye; 4 Faculty of Sport Sciences, Akdeniz University, Antalya 07600, Türkiye; 5 Vocational School of Healthy Services, Gazi University Ankara, Ankara 06490, Türkiye; 6 Faculty of Sport Sciences, Yozgat Bozok University, Yozgat 66100, Türkiye

**Keywords:** dark triad, light triad, personality, university students

## Abstract

*Background and Objectives*: The primary purpose of the article was to examine the relationship between dark and light personality traits in university students enrolled in the Faculty of Sport Sciences. *Materials and Methods*: Data were collected from 518 students (208 female, 310 male) with an average age of 20.89 (±2.25). The Short Dark Triad and the Light Triad Scales were used. Harman’s single-factor analysis minimized measurement error, and various statistical methods assessed the effect of gender and age on personality traits. *Results*: Results indicated a positive correlation among dark personality traits, while light personality traits exhibited more complex relationships. Gender and age were found to significantly influence certain personality traits. *Conclusions*: This study contributes to the sports literature by exploring the role of demographic variables in personality formation.

## 1. Introduction

Personality research in sport has a long and rich history. Recent empirical findings on athletes have revitalized this field by introducing new concepts and measurement methods. For instance, the study of personality traits in athletes has expanded significantly, offering new insights into how these traits impact performance and behavior in sports settings [1]. While personality is inherently complex, its study in sport becomes even more nuanced due to factors like success and coaching influences [1]. To understand how personality is internalized and reflected in behavior, deeper examination of psychosocial factors is needed. In the context of sport sciences, both dark triad (narcissism, Machiavellianism, and psychopathy) and light triad (humanism, Kantianism, and faith in humanity) traits may influence academic and athletic performance. Dark triad traits often manifest as manipulative and arrogant behaviors, while light triad traits are associated with positive social behaviors like empathy and fairness [2,3]. Understanding how such traits shape student attitudes and behaviors is crucial for educators and coaches.

A comprehensive review of the literature reveals that the dark triad, encompassing narcissism, Machiavellianism, and psychopathy, has been extensively studied across various populations, including athletes [4]. These traits manifest as manipulative, emotionally detached, and unethical behaviors in both social and personal interactions [5]. The former, narcissism, is characterized by an inflated sense of self-importance, a need for superiority over others, and a lack of empathy [6]. Individuals with narcissistic tendencies often overestimate their accomplishments while simultaneously underestimating those of others. Their heightened sensitivity to criticism can at times lead to conflicts in social relationships [7]. Interestingly, psychological research has found a higher prevalence of narcissism among individuals in leadership roles, suggesting a potential correlation between this trait and the attainment of elevated social status [8]. Machiavellianism, on the other hand, is marked by a willingness to manipulate others and a disregard for ethical considerations in pursuit of personal goals [9]. This trait is marked by a pragmatic approach to achieving personal goals, often involving manipulation and a disregard for conventional morality [10]. Such a strategic focus may offer advantages in competitive settings, but it may also undermine trust and damage social bonds over time [11].

Psychopathy, the last of the personality disorders mentioned, is characterized by emotional coldness, impulsivity, deficits in empathy, and a propensity for antisocial or criminal behavior [12], frequently manifesting in transgressive and unlawful acts. Psychopathic individuals exhibit a marked inability to understand or empathize with the emotions of others. This emotional detachment may facilitate a disregard for social norms, which can lead to harmful consequences within the broader context of societal expectations and legal frameworks [13]. While relatively rare in the general population, psychopathy is disproportionately represented within the criminal justice system [14].

Dark personality traits, including Machiavellianism, narcissism, and psychopathy, are characterized by socially undesirable behaviors such as manipulation, excessive competitiveness, and emotional detachment [15]. The traits have been investigated in both non-clinical [16,17] and clinical [18] populations, often assessed on a subclinical level. They shape our perception of ourselves and our environment, influencing both adaptive and maladaptive responses [19]. In the context of sports, such responses manifest as mental processes that athletes must learn and employ [20]. These processes impact various aspects of athletic performance, such as adaptation, persistence, personal satisfaction, and outcomes, ultimately shaping the athlete’s perception of success or failure.

Research in sport sciences has highlighted the significance of these traits in athletic contexts, noting that dark triad traits can both enhance and hinder performance [21]. The relationship between dark triad traits and sport sciences is significant, as these personality constructs can influence both individual athletic performance and team dynamics [21]. For example, the competitive nature inherent in narcissism and Machiavellianism may offer athletes a distinct advantage. Narcissistic athletes, driven by self-confidence and a constant need for attention, may experience heightened motivation [22]. Similarly, Machiavellian athletes may leverage their manipulative skills for strategic gain [23]. However, these traits can also undermine team cohesion and cooperation, necessitating a thorough understanding of their impact on athletes by coaches and sports professionals. Examining dark triad traits within the context of sport sciences can inform the development of effective strategies to enhance both individual and team performance.

The light triad encompasses three positive personality traits prevalent in personality psychology: humanism, Kantianism, and faith in humanity. These traits are associated with individuals exhibiting constructive, ethical, and socially beneficial behaviors [24]. Humanism refers to an individual’s tendency to be understanding and helpful towards others [25]. Humanistic individuals act with empathy and sensitivity to the emotional and physical needs of others. They are often motivated to create positive societal change and foster stronger interpersonal relationships. By promoting cooperation and collaboration, humanism plays a crucial role in maintaining social cohesion [26]. Kantianism, named after German philosopher Immanuel Kant, emphasizes unwavering adherence to ethical principles. Kantian individuals prioritize doing what is morally right, acting in accordance with moral norms and a strong sense of justice. This focus on objectivity and fairness in decision-making leads to their recognition as trustworthy and respected members of society [27]. Faith in humanity is the belief in the fundamental goodness of people and human nature. Individuals possessing this trait tend to trust others and assume positive intentions. This optimistic outlook fosters trust within social relationships and encourages individuals to take on more active and constructive roles in society, thereby strengthening social bonds [28].

Recent studies have increasingly focused on the implications of light triad traits in sports, suggesting that these traits may enhance teamwork and overall performance [29]. The light triad’s relevance to sport sciences lies in the potential impact of these personality constructs on intra-team dynamics, leadership behaviors, and overall athletic performance. Humanistic athletes, for example, may foster team cohesion and morale by demonstrating support and helpfulness towards their teammates. Kantian athletes may exemplify ethical sportsmanship through strict adherence to principles of fairness and honesty. A strong faith in humanity can contribute to a positive team spirit and an environment of mutual trust. Athletes embodying light triad characteristics may significantly enhance both individual and team performance, bolstering their psychological well-being and contributing to sustained long-term success. These traits may also inform the development of individualized approaches within sport sciences. The recent emphasis on positive personality traits in research [29,30,31] underscores the need to explore nuanced personality constructs to better understand psychological behavior in sports and complement positive psychology approaches.

The dark and light triads are not personality dimensions but rather clusters of personality traits. These constructs represent distinct combinations of characteristics that have significant implications for behavior and social interactions, particularly in competitive and cooperative settings [32]. Understanding the theoretical status of these triads is crucial for accurately interpreting their impact on individual and team dynamics in various contexts, including sports [33].

The significance of this research lies in its potential to inform the development of training methods and programs in sport sciences by tailoring them to the personality characteristics of students. Despite the growing interest in personality traits within the realm of sport sciences, there remains a notable gap in understanding how dark and light personality traits specifically impact sport sciences students. This study aims to fill this gap by examining the intricate relationships between these personality traits and their influence on both academic and athletic performance.

A deeper understanding of the personality traits of students in this field will enable the creation of more effective and individualized training approaches, ultimately enhancing their educational experience. By identifying how traits such as narcissism, Machiavellianism, psychopathy, humanism, Kantianism, and faith in humanity influence behavior and performance, educators and coaches can develop targeted strategies that foster positive traits while mitigating negative ones.

The findings of this study will contribute substantially to the existing academic literature by providing empirical data on the prevalence and impact of these traits among sport sciences students. This research will serve as a valuable reference for the development of education and training practices in sport sciences, offering insights that can lead to the optimization of training programs. By articulating the significance of this research gap and elucidating how the study addresses it, we aim to bolster the justification for this study, ultimately contributing to the advancement of educational and training methodologies in sport sciences.

The primary objective of this study is to investigate the personality traits of university students in the faculty of sport sciences, examining both their dark and light personality characteristics. Specifically, this study aims to explore the impact of demographic variables such as age, gender, career path, licensed sports status, and class level on the personality traits of students. The theoretical framework for this study is grounded in the Dual Process Model of personality traits, which posits that individuals possess both socially aversive (dark triad) and socially desirable (light triad) traits [34]. The dark triad includes Machiavellianism, narcissism, and psychopathy, which are often linked to manipulative and self-serving behaviors. In contrast, the light triad, comprising faith in humanity, humanism, and Kantianism, is associated with altruistic and ethical behaviors.

The research hypotheses are as follows, supported by relevant theories:

1. A significant correlation exists between the dark triad (Machiavellianism, narcissism, psychopathy) and light triad (faith in humanity, humanism, Kantianism) personality traits of students enrolled in the faculty of sport sciences. This hypothesis is grounded in the Dual Process Model, suggesting that individuals may exhibit a complex interplay of both positive and negative traits, which can be mutually influential [35].
2. Gender differences are expected to emerge in the dark triad and light triad personality traits of sport sciences students. This expectation is based on gender role theory, which posits that societal norms and roles shape the expression of personality traits differently in men and women [36].
3. Engaging in licensed sports is predicted to result in significant differences between the dark triad and light triad personality traits of sport sciences students. This hypothesis draws from social learning theory [37], which suggests that the structured and competitive environment of licensed sports can influence the development of both positive and negative personality traits through reinforcement and modeling.
4. Age-related differences are anticipated in the dark triad and light triad personality traits of sport sciences students. According to lifespan development theory, personality traits evolve with age due to cumulative life experiences and changing social roles [38]. It is expected that younger students may exhibit different trait profiles compared to older students, reflecting their developmental stages and life experiences.
5. Grade level is expected to create significant differences between the dark triad and light triad personality traits of sport sciences students. This hypothesis is informed by the theory of cognitive development, which posits that intellectual and moral reasoning capabilities expand with educational advancement. As students progress through their academic careers, their exposure to diverse ideas and ethical considerations may lead to variations in both dark and light personality traits [39].

By anchoring these hypotheses in established psychological theories, this study aims to provide a robust framework for understanding the complex interplay of personality traits among sport sciences students.

## 2. Materials and Methods

### 2.1. Research Model

This study employs a relational survey model to investigate the dark triad (Machiavellianism, narcissism, psychopathy) and light triad (faith in humanity, humanism, Kantianism) personality traits among sport sciences students. The primary objective is to examine the relationships between these traits and demographic variables, as well as their influence on students’ preferences within the field of sport. Data were collected from December 2023 to March 2024 via a questionnaire-based survey administered to sport sciences students at Aydın Adnan Menderes University. A random sample of 518 participants was selected for analysis, and appropriate statistical methods were employed to test the research hypotheses.

### 2.2. Participants

The current cross-sectional study included 518 sport sciences students at Aydın Adnan Menderes University (after excluding 3 participants due to missing data). The sample was selected to represent a portion of the institute’s students, ensuring a diverse and balanced representation of various sports branches. The sample consisted of 208 females (40.2%) and 310 males (59.8%), with a mean age of 20.89 years (±2.252). Participants were distributed across various sports branches, including Physical Education and Sports Teaching (20.3%), Coaching (34.7%), Sports Management (24.7%), and Recreation (20.3%). Of the participants, 55.8% expressed interest in individual sports, while 34.0% preferred team sports. Class level distribution was as follows: 1st level (44.4%), 2nd level (17.2%), 3rd level (17.0%), and 4th level (21.4%). Informed consent was obtained from all participants prior to data collection. The selection of participants aimed to capture a comprehensive overview of the student body, considering various academic levels and sports preferences (Table 1).

### 2.3. Data Collection Tools

#### 2.3.1. Short Dark Triad

The adaptation of the 27-item Short Dark Triad (SDT) scale to the Turkish language and culture was conducted by Ağralı-Ermiş et al. [40], based on the original version developed by Jones and Paulhus [41]. The scale utilizes a 5-point Likert scale, ranging from 1 (strongly disagree) to 5 (strongly agree), with items 11, 15, 17, 20, and 25 requiring reverse coding. An examination of the scale’s psychometric properties revealed a Cronbach α coefficient of 0.80 for Machiavellianism, 0.79 for narcissism, and 0.81 for psychopathy, indicating acceptable reliability.

#### 2.3.2. Light Triad Scale

The Light Triad Scale, developed by Kaufman et al. [24], consists of 12 items, with 4 items each measuring faith in humanity (1 M, 2 M, 3 M, 4 M), humanism (5 M, 6 M, 7 M, 8 M), and Kantianism (9 M, 10 M, 11 M, 12 M). Serbest and Sökmen [42] confirmed the discriminant and internal validity of the Turkish adaptation of this three-factor scale, demonstrating acceptable reliability coefficients for both sub-factors and the overall scale using Cronbach’s alpha and Gutmann coefficients. In the present study, Cronbach’s alpha coefficients were 0.83 for faith in humanity, 0.84 for humanism, and 0.83 for Kantianism, indicating strong internal consistency and reliability.

### 2.4. Data Analysis

The study utilized data obtained from the same participants during the same time period through two distinct scales designed to serve different purposes. As a notable consideration, the negative statements within the scale items were reverse-coded, which suggests the potential for common method bias (CMB)—a major source of measurement error [43]. To investigate this, the researchers applied Harman’s single-factor analysis, a widely accepted method for identifying biases that may arise from the inclusion of multiple scales in a single questionnaire completed by participants concurrently [44]. Analysis results showed that the single-factor variance accounted for 32.156% of the total variance, which indicates the absence of common method bias, as a one-factor variance of less than 50% suggests the lack of CMB [44,45]. These findings demonstrate that the measures taken to enhance the reliability of the data and the accuracy of the analyses were effective. Accordingly, the inferences drawn from the obtained results can be considered reliable and valid.

Data analysis was conducted on SPSS 25.00 and AMOS statistical software packages, with a significance level set at 0.05. Frequency, percentage, and weighted mean values were employed to examine data distribution. Normality tests, assessing skewness and kurtosis values, confirmed the normal distribution of data, allowing for the application of parametric tests. Specifically, skewness and kurtosis values for Machiavellianism (−0.209, 0.630), narcissism (−0.020, −0.110), psychopathy (0.341, −0.341), faith in humanity (−0.068, −0.136), humanism (−0.771, 0.257), and Kantianism (−0.458, 0.336) all fell within the ±1.5 range indicative of normality, as suggested by Tabachnick and Fidell [46]. To establish the validity and reliability of the scales, Cronbach’s alpha coefficient was calculated, and confirmatory factor analysis was performed. Descriptive statistics (mean and standard deviation) and correlation analysis were utilized to examine relationships between variables. *T*-tests were used for paired group comparisons, and ANOVA analysis for comparisons involving three or more groups.

## 3. Results

Before examining the hypotheses, a confirmatory factor analysis (CFA) was conducted on the AMOS 24.0 software package to test the discriminant validity of the main variables (Table 2).

The CFA results presented in Table 2 indicate that the scales have an acceptable fit for conducting analyses, and the population also has reasonable approximation errors.

Table 3 presents the descriptive statistics of all variables and the correlations between them. In general, the dark personality traits (Machiavellianism, narcissism, and psychopathy) were observed at moderate levels, while the light personality traits (faith in humanity, humanism, and Kantianism) were observed at higher levels. These findings provide an important starting point for understanding the personality profiles of the participants.

The correlation analysis (Table 3) revealed several noteworthy findings. Machiavellianism had positive and statistically significant correlations with narcissism (r = 0.279, *p* < 0.01) and psychopathy (r = 0.378, *p* < 0.01), indicating that Machiavellianism is positively associated with these other dark personality traits. Similarly, narcissism had a positive and significant correlation with psychopathy (r = 0.280, *p* < 0.01) and Machiavellianism (r = 0.279, *p* < 0.01). Psychopathy was also positively correlated with both Machiavellianism (r = 0.378, *p* < 0.01) and narcissism (r = 0.280, *p* < 0.01).

In contrast, light personality traits exhibited different relationships. No statistically significant positive or negative correlations were observed between faith in humanity and other variables (*p* > 0.05). Humanism showed positive and significant correlations with psychopathy (r = 0.470, *p* < 0.01) and Machiavellianism (r = 0.118, *p* < 0.05), but a negative and significant correlation with faith in humanity (r = −0.108, *p* < 0.05). Kantianism had positive and significant correlations with Machiavellianism (r = 0.099, *p* < 0.01) and faith in humanity (r = 0.364, *p* < 0.01). These findings suggest that dark personality traits are positively and significantly related to each other, while the relationships between light personality traits and dark traits are more complex and varied.

Table 4 presents the analysis of the differences between male and female students regarding the personality traits of the participants. The results show that male students scored higher than female students on the Machiavellianism, narcissism, and psychopathy sub-dimensions. The difference between the groups was statistically significant for the psychopathy sub-dimension, and the effect size was medium (Cohen’s d = 0.452). Conversely, female students scored higher than male students on the humanism and Kantianism sub-dimensions. These differences were also statistically significant, with noteworthy effect sizes for the humanism and Kantianism traits (Cohen’s d = 0.309 and Cohen’s d = 0.322, respectively). These findings suggest that gender has a meaningful effect on the expression of these personality traits.

Table 5 looks into the differences between the dark and light personality traits of individuals across different age groups. The results indicate that there were no statistically significant differences between the age groups in Machiavellianism, narcissism, faith in humanity, humanism, and Kantianism traits (*p* > 0.05). However, there was a significant difference between the age groups in the psychopathy trait (F(516) = 3.033, *p* < 0.05, η^2^ = 0.017). Specifically, the psychopathy trait was higher in the 18–20 age group compared to the other age groups. These findings suggest that the age variable has an effect on the psychopathy trait, while the other personality traits examined did not differ significantly based on age.

Table 6 analyzes the differences in dark and light personality traits among individuals with varying career preferences. The results indicate that there were no statistically significant differences between career preference groups in Machiavellianism, narcissism, psychopathy, faith in humanity, humanism, and Kantianism traits (*p* > 0.05). This suggests that individuals with different career preferences generally have similar personality trait profiles. Furthermore, when examining the effect sizes of the analyses, very low effect sizes were obtained for the dark and light personality traits (η^2^ < 0.01). This indicates that the effect of career preferences on personality traits is negligible. Together, these findings demonstrate that the participants’ personality traits are not strongly associated with their chosen career paths.

Table 7 compares the personality characteristics of individuals with different sport preferences. According to the results of the analyses, there was no statistically significant difference between the groups (*p* > 0.05).

Table 8 presents the results of analyses examining differences between class levels in terms of Machiavellianism, narcissism, psychopathy, faith in humanity, humanism, and Kantianism. Significant differences were observed for psychopathy (F = 4.682, *p* < 0.05, η^2^ = 0.027) and faith in humanity (F = 3.183, *p* < 0.05, η^2^ = 0.018), indicating that students’ scores on these traits vary according to their grade level. The effect size for psychopathy was large, while the effect size for faith in humanity was moderate. No significant differences were found for Machiavellianism (F = 2.422, *p* > 0.05, η^2^ = 0.014), narcissism (F = 0.442, *p* > 0.05, η^2^ = 0.003), humanism (F = 2.301, *p* > 0.05, η^2^ = 0.013), or Kantianism (F = 1.472, *p* > 0.05, η^2^ = 0.009), suggesting that these traits are relatively consistent across different grade levels. These findings underscore the potential influence of academic progression on specific personality traits among sport sciences students.

## 4. Discussion

This study examined the relationship between dark and light personality traits among sport sciences students, as well as the influence of demographic variables on these traits. The findings contribute to a deeper understanding of the complex and diverse personalities within this population, informing future research directions.

Correlational analyses revealed relationships among dark and light personality traits consistent with established academic literature, highlighting their prevalence among students. The moderate levels of Machiavellianism, narcissism, and psychopathy, along with their significant positive correlations, support the notion that these dark traits can coexist [47]. This aligns with previous research suggesting shared mechanisms underlying these traits, leading to behavioral tendencies such as social manipulation and lack of empathy [41]. The significant positive relationship between Machiavellianism and narcissism (r = 0.279, *p* < 0.01), as well as between Machiavellianism and psychopathy (r = 0.378, *p* < 0.01), indicates that manipulative tendencies often accompany self-centeredness and low emotional reactivity [48]. These relationships underscore the interconnected nature of dark personality constructs [4]. For instance, recent research on Spanish athletes has revealed strong interrelationships among dark triad traits in competitive sports environments [49]. Narcissism was associated with both the desire to win and the fear of losing, Machiavellian tendencies were heightened when athletes felt defeated, and psychopathic tendencies were linked to feelings of inferiority and fear of failure. Such findings suggest that dark personality traits are not solely individualistic but also influenced by psychological responses and self-perceptions within competitive sports contexts [49].

In contrast, the absence of significant relationships or negative correlations between light triad traits like faith in humanity and humanism with dark triad traits indicates distinct social tendencies between these two clusters [50]. Although the positive correlation between humanism and psychopathy (r = 0.470, *p* < 0.01) appears contradictory, it may reflect the strategic use of humanistic tendencies by manipulative individuals to facilitate social connections. Significant positive relationships between Kantianism and Machiavellianism (r = 0.099, *p* < 0.01) and between Kantianism and faith in humanity (r = 0.364, *p* < 0.01) may suggest a link between moral values and social understanding and cooperation. However, these relatively weak relationships highlight the complex interplay of intellectual personality traits within social contexts.

Analysis of the gender variable revealed that male students scored higher in Machiavellianism, narcissism, and psychopathy, while female students scored higher in humanism and Kantianism, which seems to in accord with previous research on gender differences in personality traits. Similar patterns of higher scores for males in the dark triad traits have been reported [51,52]. The large effect size observed for the psychopathy sub-dimension among male students is consistent with previous findings [53,54]. The elevated dark triad scores in males may be attributed to social pressures and gender role expectations that encourage competitive and aggressive behaviors. The significant difference observed in psychopathy, in particular, may be explained by the tendency for males to exhibit more aggressive or risk-taking behaviors in line with societal norms.

Conversely, the higher scores of female students in humanism and Kantianism support previous research demonstrating that women generally exhibit higher levels of empathy and cooperation [55]. Significant differences in these traits suggest a greater propensity for prosocial tendencies among women [56]. Societal expectations emphasizing empathy, caregiving, and social responsibility may contribute to women’s higher scores on light personality traits.

Analysis of the age variable revealed a significant difference between age groups in psychopathy, suggesting this trait may vary with age and be more pronounced in young adulthood (18–20 years), which coincides well with the relevant literature indicating potential developmental changes in personality traits over time. The observed difference in psychopathy suggests that psychopathic tendencies, such as risk-taking, lack of empathy, and emotional coldness, may be more prominent in younger individuals [57]. This could be attributed to the incomplete development of the brain’s anterior cortex during adolescence and early adulthood, potentially leading to impulsivity and reduced emotional control [58].

However, the absence of significant differences between age groups in Machiavellianism, narcissism, faith in humanity, humanism, and Kantianism suggests greater stability of these traits across different ages. In particular, the stability of narcissism supports previous research indicating its enduring nature [59]. The consistent levels of light triad traits across age groups align with the hypothesis that adherence to general moral and social norms remains relatively constant throughout adulthood [60], potentially due to the establishment of moral foundations during childhood. Overall, the age-related changes observed in psychopathy highlight its potential risk for the psychosocial development of young adults. The stability of other personality traits suggests a more enduring structure that persists across different age groups. These findings provide a foundation for further investigation into the influence of age on specific dark and light triad traits. While the analysis included all age groups present in the sample, it is important to note the relatively small sample sizes for the 24–26 (*n* = 20) and 27 and over (*n* = 7) age groups. These small sizes may limit the generalizability of the findings for these specific age groups. Future research should aim to include larger samples for these age groups to validate the observed trends and provide more robust conclusions.

A deeper look into the career preference variable revealed no significant effect on personality traits. Furthermore, the minimal effect size suggests that career preferences have a negligible influence on personality. These results support the previous work in the literature highlighting the complex relationship between career choices and personality traits. Recent research has found that dark personality traits, such as Machiavellianism, narcissism, and psychopathy, do not significantly impact career preferences [61], which suggests that these traits do not influence individuals’ inclinations towards specific career paths and may be equally prevalent across various fields. Similarly, the absence of a significant effect of career preferences on light personality traits, such as faith in humanity, humanism, and Kantianism, implies that these traits are rooted in an individual’s fundamental value system, independent of professional development [62]. Moral and social values may demonstrate internal consistency regardless of occupation.

While certain career paths emphasizing social skills, such as leadership positions, may attract individuals with Machiavellian or narcissistic tendencies [4], the current findings do not support a clear distinction between career preferences and overall personality traits. However, it is important to acknowledge that career paths may indirectly influence personality traits over time. Further research is needed to explore this potential interaction.

Evaluation of the sport preference variable revealed no significant effect on personality traits, suggesting that individual personality structures remain consistent regardless of sport choice. These findings indicate that athletes do not exhibit distinct personality profiles based on their chosen sport, and that personality traits may be influenced by factors other than sport preference. This outcome corroborates the relevant literature elucidating the intricate interplay between sport preferences and personality traits. Furthermore, research suggests that dark personality traits observed in athletes are not associated with specific sports but rather with individual competitive attitudes and personal ambitions [63]. Traits like Machiavellianism and narcissism can manifest as achievement orientation and self-confidence in competitive environments, yet these tendencies may be equally prevalent across different sports. Likewise, the consistent expression of light personality traits among athletes from various sports suggests a shared foundation of social integration and prosocial behaviors [64]. Team sports, in particular, foster the development of cooperation, solidarity, and empathy through mutual learning and interaction.

Upon scrutinizing the findings concerning the grade level variable, statistically significant disparities emerged in the dimensions of psychopathy and faith in humanity, suggesting these traits may be influenced by academic progression. The observed variation in psychopathy aligns with the notion that increased social exposure and maturation associated with higher grades may mitigate psychopathic tendencies like emotional volatility and lack of empathy [65]. This is supported by research indicating a decrease in such tendencies with age [57,66,67], implying that students may adjust their psychopathic inclinations in response to evolving social contexts as they advance through their education. The observed difference in faith in humanity further highlights the potential for students’ worldviews and beliefs to transform with academic advancement. Exposure to diverse social environments and intellectual stimuli in higher grades may foster a more nuanced understanding of human nature [68]. This finding suggests that grade level may play a role in shaping faith in humanity. The absence of significant differences in Machiavellianism, narcissism, humanism, and Kantianism across grade levels indicates relative stability of these traits, independent of educational influences [69]. In particular, traits like narcissism, which play a fundamental role in personal values and self-perception, may exhibit enduring stability across educational stages.

### Limitations of the Research

While this study provides valuable insights into the relationship between dark and light personality traits among sport sciences students and the influence of demographic variables, several limitations should be noted:

- Sample Size and Generalizability: The sample consisted exclusively of sport sciences students, which may limit the generalizability of the findings to other populations. Future research should include a more diverse sample to enhance the applicability of the results.
- Cross-sectional Design: This study employed a cross-sectional design, which restricts the ability to infer causality between personality traits and demographic variables. Longitudinal studies are needed to establish causal relationships and examine changes in these traits over time.
- Self-report Measures: The reliance on self-report questionnaires may introduce response biases, such as social desirability or self-deception. Employing multiple methods of data collection, including behavioral assessments and peer reports, could provide a more comprehensive understanding of personality traits.
- Cultural Context: This study was conducted within a specific cultural context, which may influence the expression and interpretation of personality traits. Cross-cultural comparisons would help to determine the universality of the findings and account for cultural variations in personality.
- Limited Scope of Personality Traits: While this study focused on dark and light triad traits, it did not consider other relevant personality dimensions, such as the Big Five traits. Including a broader range of personality characteristics could offer a more holistic view of the participants’ personalities.
- Interaction Effects: Although this study examined the influence of individual demographic variables, it did not explore potential interaction effects between variables such as gender and age. Future research should investigate these interactions to provide a more nuanced understanding of the determinants of personality traits.
- Contextual Factors: This study did not account for contextual factors such as academic performance, social environment, or extracurricular activities, which may influence the development and expression of personality traits. Considering these factors in future research could provide additional insights.

## 5. Conclusions

This study investigated the relationship between dark and light personality traits among sport sciences students and the influence of demographic variables on these traits. The findings provide significant insights into these relationships, as well as their broader implications for academic and athletic contexts:

- ***Correlational Analyses***: The correlational analyses revealed significant relationships among dark personality traits, confirming the co-existence of Machiavellianism, narcissism, and psychopathy within the student population. Machiavellianism was positively correlated with both narcissism and psychopathy. These findings are consistent with the established literature, supporting the hypothesis that dark traits often co-occur due to shared underlying mechanisms. In contrast, the absence of significant relationships or negative correlations between dark and light personality traits underscores the distinct social tendencies inherent to these clusters. The positive correlation between humanism and psychopathy may reflect the strategic use of humanistic tendencies by manipulative individuals to facilitate social connections. Additionally, significant positive relationships between Kantianism and Machiavellianism and between Kantianism and faith in humanity suggest a link between moral values and social understanding and cooperation, although these relationships are relatively weak, highlighting the complex interplay of intellectual personality traits within social contexts.
- ***Gender Differences***: The analysis of gender differences indicated that male students scored higher in Machiavellianism, narcissism, and psychopathy, while female students scored higher in humanism and Kantianism. These results align with previous research on gender differences in personality traits, confirming the hypothesis that gender plays a significant role in the expression of both dark and light traits. The effect size for psychopathy among male students was notably large, which is consistent with the literature on gender-specific behavioral tendencies.
- ***Age Group Comparisons***: This study found a significant difference in psychopathy across different age groups, with higher scores observed in the 18–20 age group. This supports the hypothesis that psychopathic tendencies are more pronounced in younger adults, likely due to developmental factors such as the incomplete maturation of the anterior cortex. However, no significant differences were found in Machiavellianism, narcissism, faith in humanity, humanism, and Kantianism across age groups, indicating stability in these traits over time. The relatively small sample sizes for the 24–26 and 27 and over age groups may limit the generalizability of these specific findings.
- ***Career Preference***: The analysis showed no significant effect of career preferences on personality traits, with minimal effect sizes suggesting that career choices have a negligible influence on the expression of both dark and light traits. This finding is in line with the hypothesis that personality traits are rooted in fundamental value systems, independent of professional development.
- ***Sport Preference***: The results indicated no significant differences in personality traits based on sport preferences. This suggests that athletes do not exhibit distinct personality profiles based on their chosen sport, supporting the hypothesis that personality traits are influenced by individual competitive attitudes and personal ambitions rather than the specific sport.
- ***Grade Level***: Significant disparities were found in the dimensions of psychopathy and faith in humanity across different grade levels. Higher grades were associated with lower psychopathic tendencies, likely due to increased social exposure and maturation. Conversely, faith in humanity appeared to increase with academic progression, suggesting that educational experiences may enhance positive social outlooks. The stability of Machiavellianism, narcissism, humanism, and Kantianism across grade levels indicates that these traits remain relatively constant throughout academic development.

In conclusion, this study confirmed several hypotheses regarding the influence of demographic variables on dark and light personality traits, while also highlighting areas where further research is necessary to fully understand these complex relationships.

### Recommendations

The findings of this study offer valuable insights into the relationship between dark and light personality traits among sport sciences students and their association with specific demographic variables. To further elucidate this area and provide practical utility, future research and practitioners may consider the following recommendations:

- ***Cross-cultural Comparisons***: Conduct comparative analyses of sport students in different countries to understand how cultural norms influence the development and expression of dark and light personality traits. This can help tailor interventions to specific cultural contexts, enhancing their effectiveness.
- ***Sport-specific Analysis***: Examine the differences in dark and light personality traits among athletes in various sports. This can inform the development of sport-specific training and support programs that address the unique psychological demands and cultural dynamics of each sport.
- ***Longitudinal Studies***: Implement longitudinal studies to track the development of personality traits over time. Understanding the temporal changes in traits like psychopathy can guide the design of long-term support and intervention programs that adapt to the evolving needs of athletes.
- ***Age-specific Investigations***: Investigate the development of personality traits across different age groups. This can help in creating age-appropriate interventions and support systems that address the specific challenges faced by athletes at various stages of their careers.
- ***Intervention and Support Programs***: Develop and implement educational interventions and psychosocial support programs tailored to students exhibiting dark personality traits. For instance, cognitive behavioral therapy (CBT) techniques can be used to address manipulative or aggressive behaviors, while programs promoting empathy and cooperation can enhance light personality traits.
- ***Mentorship Programs***: Establish mentorship programs where experienced athletes or coaches guide younger athletes, emphasizing the development of positive traits such as humanism and Kantianism. Mentors can model ethical behavior and provide support in managing negative traits.
- ***Gender Role Exploration***: Further research is needed to explore how gender roles and societal expectations influence the development and expression of dark and light personality traits. Practitioners can use this information to create gender-sensitive interventions that address specific needs and challenges faced by male and female athletes.
- ***Training Workshops***: Organize workshops for coaches and educators to raise awareness about the impact of personality traits on student behavior and performance. These workshops can provide strategies for managing negative traits and fostering positive ones, ultimately creating a more supportive and effective training environment.
- ***Peer Support Groups***: Establish peer support groups where athletes can share their experiences and strategies for managing dark traits and enhancing light traits. Such groups can provide a sense of community and mutual support, promoting psychological well-being.

By implementing these specific strategies and interventions, practitioners can effectively mitigate the negative implications of dark personality traits and enhance the positive impact of light personality traits on student attitudes and behaviors. These actionable steps not only enhance the practical utility of the research findings but also contribute to the overall development and success of sport sciences students.

## Figures and Tables

**Table 1:** Exploring the influence of dark triad and light triad traits on sport sciences students.

	*N*	*%*
**Gender**		
Female	208	40.2
Male	310	59.8
**Age Category**		
18–20	270	52.1
21–23	221	42.7
24–26	20	3.9
27 and over	7	1.4
**Career Preferences**		
Physical Education and Sports Teaching	105	20.3
Coaching	180	34.7
Sports Management	128	24.7
Recreation	105	20.3
**Sport Type**		
Individual Sports	289	55.8
Team Sports	176	34.0
Not Engaged in Licensed Sport	53	10.2
**Class**		
1st Level	230	44.4
2nd Level	89	17.2
3rd Level	88	17.0
4th Level	111	21.4

**Table 2:** Goodness of fit index.

Model	χ^2^ (df)	χ^2^/df	Δχ^2^ (df)	CFI	RMR	GFI	RMSEA
Dark Triad	396.383 (59)	1.659	2104.184 (71)	0.94	0.075	0.95	0.036
Light Triad	82.922 (41)	2.022	1827.534 (66)	0.97	0.054	0.97	0.044

Note: χ^2^: Chi-square; Δχ^2^: delta Chi-square; df: degree of freedom; CFI: Comparative Fit Index; RMR: Root Mean Square Residual; GFI: Goodness of Fit Index; RMSEA: Root Mean Square Error of Approximation.

**Table 3:** Descriptive statistics and correlations between all variables.

	M	Sd.	1	2	3	4	5	6
1. Machiavellianism	3.29	0.637	-					
2. Narcissism	3.31	0.640	0.279 **	-				
3. Psychopathy	2.58	0.744	0.378 **	0.280 **	-			
4. Faith in Humanity	3.24	0.873	0.032	0.002	−0.064	-		
5. Humanism	4.04	0.838	0.118 **	0.063	0.470 **	−0.108 *	-	
6. Kantianism	3.73	0.758	0.099 **	0.025	−0.068	0.364 **	0.502 **	-

Note: * *p* < 0.05, ** *p* < 0.01; M: mean, Sd.: standard deviation.

**Table 4:** Comparison of dark triad and light triad sub-dimensions by gender.

Variables	Female		Male		t	*p*	Cohen’s d
	x	SD	x	SD			
**Machiavellianism**	3.240	0.608	3.331	0.654	−1.598	0.111	0.144
**Narcissism**	3.280	0.649	3.334	0.635	−929	0.354	0.084
**Psychopathy**	2.384	0.745	2.714	0.714	−5.060	0.000 **	0.452
**Faith in Humanity**	3.314	0.840	3.199	0.892	1.480	0.140	0.133
**Humanism**	4.199	0.764	3.946	0.870	3.409	0.001 **	0.309
**Kantianism**	3.878	0.694	3.639	0.785	3.556	0.000 **	0.322

Notes: ** *p* < 0.01. x = mean; SD = standard deviation; female (*n* = 208); male (*n* = 310); Cohen’s d = effect size.

**Table 5:** Comparison of dark triad and light triad sub-dimensions by age groups.

Variables	18–20		21–23		24–26		27 and Over		F	η^2^
	x	SD	x	SD	x	SD	x	SD		
**Machiavellianism**	3.255	0.622	3.333	0.656	3.483	0.542	3.063	0.780	1.519	0.009
**Narcissism**	3.063	0.780	3.372	0.638	3.227	0.498	3.428	0.486	1.299	0.008
**Psychopathy**	2.527	0.713	2.671	0.783	2.538	0.627	2.000	0.584	3.033 *	0.017
**Faith in Humanity**	3.248	0.868	3.213	0.884	3.275	0.711	4.071	0.898	2.207	0.013
**Humanism**	4.047	0.859	4.056	0.817	3.937	0.826	4.107	0.814	0.135	0.001
**Kantianism**	3.713	0.788	3.753	0.732	3.800	0.700	3.857	0.659	0.228	0.001

Notes: * *p* < 0.05; x = mean; SD = standard deviation; 18–20 (*n* = 270); 21–23 (*n* = 221); 24–26 (*n* = 20); 27 and over (*n* = 7); η^2^ = effect size.

**Table 6:** Comparison of dark and light triad sub-dimensions by career preferences.

Variables	Physical Education and Sports Teaching		Coaching		Sport Management		Recreation		F	η^2^
	x	SD	x	SD	x	SD	x	SD		
**Machiavellianism**	3.387	0.610	3.273	0.627	3.302	0.638	3.230	0.677	1.165	0.007
**Narcissism**	3.305	0.608	3.324	0.646	3.303	0.632	3.311	0.678	0.034	0.000
**Psychopathy**	2.574	0.787	2.540	0.733	2.620	0.777	2.613	0.679	0.371	0.002
**Faith in Humanity**	3.273	0.862	3.252	0.858	3.310	0.827	3.126	0.961	0.930	0.005
**Humanism**	4.054	0.810	4.030	0.856	4.146	0.754	3.950	0.925	1.096	0.006
**Kantianism**	3.816	0.620	3.695	0.804	3.744	0.730	3.711	0.836	0.603	0.004

Notes: x = mean; SD = standard deviation; Physical Education and Sports Teaching (*n* = 105); Coaching (*n* = 180); Sport Management (*n* = 128); Recreation (*n* = 105); η^2^ = effect size.

**Table 7:** Comparison of dark triad and light triad sub-dimensions by sport type.

Variables	Team Sports		Individual Sports		Not Doing Licensed Sport		F	η^2^
	x	SD	x	SD	x	SD		
**Machiavellianism**	3.326	0.625	3.218	0.670	3.379	0.576	2.085	0.008
**Narcissism**	3.303	0.639	3.338	0.653	3.276	0.613	0.258	0.001
**Psychopathy**	2.559	0.730	2.661	0.756	2.440	0.761	2.112	0.008
**Faith in Humanity**	3.224	0.872	3.261	0.880	3.306	0.868	0.238	0.001
**Humanism**	4.077	0.834	3.975	0.858	4.127	0.785	1.063	0.004
**Kantianism**	3.739	0.745	3.723	0.800	3.754	0.697	0.045	0.000

Notes: x = mean; SD = standard deviation; Team Sports (*n* = 289); Individual Sports (*n* = 176); Not Doing Licensed Sport (*n* = 53); η^2^ = effect size.

**Table 8:** Comparison of dark triad and light triad sub-dimensions by grade level.

Variables	1st Level		2nd Level		3rd Level		4th Level		F	η^2^
	x	SD	x	SD	x	SD	x	SD		
**Machiavellianism**	3.237	0.624	3.232	0.577	3.369	0.656	3.404	0.681	2.422	0.014
**Narcissism**	3.287	0.659	3.330	0.591	3.292	0.651	3.367	0.635	0.442	0.003
**Psychopathy**	2.494	0.711	2.518	0.690	2.596	0.838	2.803	0.735	4.682 *	0.027
**Faith in Humanity**	3.241	0.887	3.168	0.795	3.485	0.875	3.126	0.876	3.183 *	0.018
**Humanism**	4.032	0.881	3.971	0.798	4.252	0.712	3.977	0.854	2.301	0.013
**Kantianism**	3.687	0.817	3.764	0.613	3.700	0.741	3.735	0.744	1.472	0.009

Notes: * *p* < 0.05; x = mean; SD = standard deviation; 1st Level (*n* = 230); 2nd Level (*n* = 89); 3rd Level (*n* = 88); 4th Level (*n* = 111); η^2^ = effect size.

## Data Availability

Data supporting the findings of this study are available from the corresponding author upon reasonable request.
